# Role of the Ubiquitin Proteasome System in Regulating Skin Pigmentation

**DOI:** 10.3390/ijms10104428

**Published:** 2009-11-20

**Authors:** Hideya Ando, Masamitsu Ichihashi, Vincent J. Hearing

**Affiliations:** 1 Skin Aging and Photo-aging Research Center, Doshisha University, Kizugawa, Kyoto 619-0225, Japan; E-Mails: hideyaando@aol.com (H.A.); mm_ichihashi@hotmail.com (M.I.); 2 Kobe Skin Research Institute, Kobe, Hyogo 650-0047, Japan; 3 Laboratory of Cell Biology, National Cancer Institute, National Institutes of Health, Bethesda, MD 20892, USA

**Keywords:** fatty acid, melanin, melanocyte, tyrosinase, skin, pigmentation

## Abstract

Pigmentation of the skin, hair and eyes is regulated by tyrosinase, the critical rate-limiting enzyme in melanin synthesis by melanocytes. Tyrosinase is degraded endogenously, at least in part, by the ubiquitin proteasome system (UPS). Several types of inherited hypopigmentary diseases, such as oculocutaneous albinism and Hermansky-Pudlak syndrome, involve the aberrant processing and/or trafficking of tyrosinase and its subsequent degradation which can occur due to the quality-control machinery. Studies on carbohydrate modifications have revealed that tyrosinase in the endoplasmic reticulum (ER) is proteolyzed via ER-associated protein degradation and that tyrosinase degradation can also occur following its complete maturation in the Golgi. Among intrinsic factors that regulate the UPS, fatty acids have been shown to modulate tyrosinase degradation in contrasting manners through increased or decreased amounts of ubiquitinated tyrosinase that leads to its accelerated or decelerated degradation by proteasomes.

## 1. Introduction

Solar ultraviolet (UV)-B radiation (290–320 nm) is absorbed by DNA in the epidermis while UV-A radiation (320–400 nm) generates reactive oxygen species in the dermis. Both types of UV radiation damage DNA directly or indirectly, which can lead to the formation of mutations, which can in turn result in UV-induced skin cancers. In the basal layer of the epidermis, there are specialized cells named melanocytes, that produce melanins. The role of those cells and the melanins is to prevent UV-induced skin cancers by absorbing the UV energy and thus protecting against nuclear DNA damage. Melanin synthesis in the skin, hair and eyes is ultimately regulated by tyrosinase, the critical rate-limiting enzyme produced by melanocytes within those tissues. Following the translation and subsequent processing of tyrosinase in the ER and Golgi, it is trafficked to specialized organelles, termed melanosomes, wherein melanin is synthesized and deposited. In the skin and hair, melanosomes are transferred from melanocytes to neighboring keratinocytes and are distributed in those tissues to produce visible color.

Although melanin is important for photoprotection from UV radiation, excess melanin production and/or its abnormal distribution can cause irregular hyperpigmentation of the skin, such as occurs in melasma and in age spots, and those types of lesions are often of cosmetic and/or psychological concern to men and women. In order to develop therapies or prophylactics that improve or prevent hyperpigmentary disorders, disruption of tyrosinase activity has usually been targeted. To date, many approaches that can inhibit tyrosinase activity and thus decrease melanin production have been reported, for example, the inhibition of tyrosinase mRNA transcription, the disruption of tyrosinase glycosylation, the competitive or non-competitive inhibition of tyrosinase catalytic activity, or the acceleration of tyrosinase degradation, all of which would reduce melanin synthesis and deposition [1]. However, relatively little attention has been paid to regulating pigmentation via modulation of the stability of tyrosinase, which depends on tyrosinase processing and maturation in the ER and Golgi, and its degradation via the ubiquitin proteasome system (UPS) and/or the endosomal/lysosomal system.

## 2. Role of the UPS in Melanin Synthesis

**Scheme 1.**
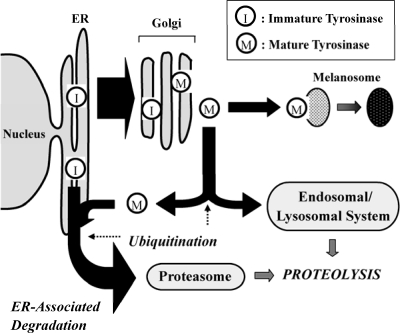
Scheme depicting the processing and degradation of tyrosinase within melanocytes. After maturation in the Golgi, tyrosinase is trafficked either to melanosomes for melanin synthesis or to the degradation machinery. The proteolysis of tyrosinase is divided into two pathways, that is, one integrated into the ERAD in the UPS whereas the other is integrated into the endosomal/lysosomal degradation system. Whether mature tyrosinase that integrates into the ERAD transports back to the ER or bypasses the ER is still unknown. In addition, the intracellular location where the ubiquitination of tyrosinase occurs remains to be determined (adapted from Figure 3 of [1]).

Levels of intracellular proteins are regulated by the balance between their synthesis and degradation, which is also true for tyrosinase. However, in contrast to effects on other proteins, the reduced stability and decreased function of tyrosinase has dramatic results on ensuing pigmentation. Early studies on the stability of tyrosinase revealed that tyrosinase is degraded endogenously in melanoma cells [2]. Later studies on tyrosinase degradation revealed that a variety of intrinsic factors in the epidermis have the potency to increase tyrosinase degradation, *e.g.*, TGF-β1, TNF-α and linoleic acid [3–5]. Although the endogenous degradation of tyrosinase was first observed several decades ago, the specific mechanism(s) that regulates tyrosinase degradation had not been clarified until the UPS was found to be involved in that process [6]. Studies on carbohydrate modifications of tyrosinase have revealed that tyrosinase destined for degradation in the ER is proteolyzed by proteasomes via ER-associated protein degradation (ERAD) [7,8]. ERAD is a mechanism for quality-control which involves retention in the ER and retro-translocation into the cytosol of misfolded or unassembled secretory proteins followed by their deglycosylation, ubiquitination and subsequent proteolysis by proteasomes [9–11]. Recently, it was reported that tyrosinase degradation can also occur following its complete maturation in the Golgi, which suggests that tyrosinase is also subject to post-Golgi-associated protein degradation [12–14] (see Scheme 1). The relative contributions of UPS and the endosomal /lysosomal system in the degradation pathway of mature tyrosinase is not yet known.

Oculocutaneous albinism type 1 is an autosomal recessive disease caused by mutations in the gene encoding tyrosinase which result in a deficiency of pigmentation of the skin, hair and eyes [15,16]. In albino melanocytes or in amelanotic melanoma cells, the aberrant retention of tyrosinase in the ER and its subsequent degradation occur owing to the quality-control machinery, indicating that oculocutaneous albinism, at least in part, is an ER retention disease [6,16,17]. Molecular chaperones in the ER which assist protein maturation, such as calnexin and calreticulin, play roles in the retention of misfolded proteins in the ER [18]. In fact, mutations in tyrosinase enhance and prolong its association with calnexin and this in turn causes the retention of mutant tyrosinase in the ER [16] after which it is degraded by ERAD coincident with its dissociation from ER chaperones [19].

The oligosaccharide trimming of sugar chains plays a pivotal role in the targeting of tyrosinase to the cytosol for degradation via ERAD [20]. This is also supported by the finding that when calnexin binding to the glucosylated *N*-glycans of tyrosinase is disrupted by inhibition of α-glucosidase I, an inhibitor of the ER-processing enzymes, tyrosinase escapes from ERAD to melanosomes in a misfolded form that has a normal half-life [21]. Once the tyrosinase:calnexin complex has formed, inhibition of α-glucosidase II prevents tyrosinase from being released spontaneously from the complex, which results in its incorrect folding and subsequent degradation [22]. Furthermore, it has been demonstrated that ubiquitination is required for the retro-translocation of ER glycoproteins to the cytosol for degradation by proteasomes [23]. Together with the importance of ubiquitination in the ERAD system, it has also been reported that ubiquitination plays a role as a sorting determinant for entry into the endosomal degradative system [24]. Thus carbohydrate modification, molecular chaperone engagement and ubiquitination all play pivotal roles in regulating the degradation and stability of tyrosinase.

## 3. Fatty Acid-Induced Regulation of the UPS on Tyrosinase

Various physiological and non-physiological factors that regulate the UPS have been reported. For example, dramatic activation of proteasomes can be induced by various treatments *in vitro*, including incubation with basic polypeptides, sodium dodecyl sulfate, guanidine HCl, amino acids or fatty acids, whereas glycerol helps maintain proteasome activity in the latent form [25–29]. Regarding fatty acid-induced regulation of the UPS, the proteolytic activity of 20S proteasomes is increased by physiological concentrations of fatty acids, such as oleic acid and linoleic acid, in rat skeletal muscle [30] or spinach leaves [31]. Moreover, it was reported that 15(*S*)-hydroxyeicosatetraenoic acid increased expression of the regulatory components of the UPS, *i.e.*, the proteasome subunit and E2 ubiquitin-conjugating enzymes, possibly through the intervention of NF-κB, and this process could be inhibited by eicosapentaenoic acid [32]. Recently, it was also reported that fatty acids, such as palmitic acid and oleic acid, strongly enhance CD36 ubiquitination while insulin reduces it [33].

Among intrinsic factors that regulate the UPS, fatty acids are major components of biological cell membranes that play important roles in intracellular signaling and as precursors for ligands that bind to nuclear receptors [34–36]. Fatty acids have been shown to regulate tyrosinase degradation in contrasting manners via their effects on the UPS, that is, linoleic acid (unsaturated fatty acid, C18:2) accelerates whereas palmitic acid (saturated fatty acid, C16:0) decelerates, the degradation of tyrosinase [5]. Further study showed that fatty acids regulate, that is, linoleic acid increases, while palmitic acid decreases, the ubiquitination of tyrosinase which leads in turn to the accelerated or decelerated degradation of tyrosinase by proteasomes, respectively [37]. As for the pharmacological efficiency of linoleic acid on the skin, topical application of linoleic acid has been shown to decrease UV-induced hyperpigmentation of the skin that leads to the prevention of hyperpigmentary disorders [38,39], showing that a specific regulator of the UPS could be useful for a topical drug. In broader terms, the UPS-mediated protein degradation of membrane glycoproteins including tyrosinase might be regulated physiologically, at least in part, by fatty acids.

## 4. Conclusions

Pigmentation of the skin plays important roles, particularly in preventing UV-induced skin cancers. Degradation of tyrosinase by the UPS is a critical regulatory point for modulating skin pigmentation. The physiological factors that regulate the UPS, *e.g.*, fatty acids and amino acids, may prove to be useful for effective skin lightening cosmetics based on the regulatory mechanism of tyrosinase degradation.
